# Role of TNF-Alpha, IFN-Gamma, and IL-10 in the Development of Pulmonary Tuberculosis

**DOI:** 10.1155/2012/745483

**Published:** 2012-11-28

**Authors:** Yone Vila Nova Cavalcanti, Maria Carolina Accioly Brelaz, Juliana Kelle de Andrade Lemoine Neves, José Candido Ferraz, Valéria Rêgo Alves Pereira

**Affiliations:** ^1^Departamento de Biologia, UFRPE, Dom Manoel de Medeiros, S/N, Dois Irmãos, 52171-900 Recife, PE, Brazil; ^2^Departamento de Imunologia, Centro de Pesquisas Aggeu Magalhães (CPqAM/FIOCRUZ), Avenida Professor Moraes Rego S/N, Cidade Universitária, Campus da UFPE, 50670-420 Recife, PE, Brazil; ^3^Núcleo de Educação Física e Ciências do Esporte, Centro Acadêmico de Vitória, Universidade Federal de Pernanbuco (UFPE), Vitória de Santo Antão, PE, Brazil

## Abstract

Host immune response against *Mycobacterium tuberculosis* is mediated by cellular immunity, in which cytokines and Th1 cells play a critical role. In the process of control of the infection by mycobacteria, TNF-alpha seems to have a primordial function. This cytokine acts in synergy with IFN-gamma, stimulating the production of reactive nitrogen intermediates (RNIs), thus mediating the tuberculostatic function of macrophages, and also stimulating the migration of immune cells to the infection site, contributing to granuloma formation, which controls the disease progression. IFN-gamma is the main cytokine involved in the immune response against mycobacteria, and its major function is the activation of macrophages, allowing them to exert its microbicidal role functions. Different from TNF-alpha and IFN-gamma, IL-10 is considered primarily an inhibitory cytokine, important to an adequate balance between inflammatory and immunopathologic responses. The increase in IL-10 levels seems to support the survival of mycobacteria in the host. Although there is not yet conclusive studies concerning a clear dichotomy between Th1 and Th2 responses, involving protective immunity and susceptibility to the disease, respectively, we can suggest that the knowledge about this responses based on the prevailing cytokine profile can help to elucidate the immune response related to the protection against *M. tuberculosis*.

## 1. Introduction

The genus *Mycobacterium* displays more than 100 known species, with a broad geographic distribution, habitat diversity, and diverse relations with other organisms, including more than 20 species presenting different degrees of pathogenicity to humans [1]. *Mycobacterium tuberculosis* (*M. tuberculosis*) (MTB), an intracellular facultative bacillus, is the most frequent species isolated in human tuberculosis (TB) cases.

Pulmonary tuberculosis is a global public health problem, presenting high incidence in Brazil. It is still the world's leading cause of death from a single infectious agent. Most infections are asymptomatic and latent however around 5% to 10% of infected people progress to the disease development at each year, pulmonary tuberculosis being found in most cases. Each second a person is infected with *M. tuberculosis* in the world. From the lungs, the organism is efficiently transmissible through aerosol. It is estimated that, on average, a person with active TB can infect between 10 and 15 individuals per year [2]. The World Health Organization (WHO) works intensively to significantly reduce the TB cases and halve this disease death number until 2015 [3]; however, the number of multidrug-resistant TB cases is rising, and this increasingly compromises the disease control [4, 5].

Latent TB is defined as an infection with *M. tuberculosis* that remains within macrophages without replicate, but that retains the ability to exit latency and cause active disease when there is an interruption of the protective immune response. The reactivation of a latent infection requires the activation of the quiescent bacilli. Several factors can trigger the development of active disease from reactivation of latent infection, which usually involves the decline of the immune response. HIV infection is the most important risk factor for progression to active disease due to depletion of CD4^+^ T cells [6]. Advanced age, malnutrition, and medical conditions that compromise the immune system are also risk factors for the reactivation [7, 8].

Tuberculosis progression is associated with the immune *status*. It is known that host protective immune response against this pathogen is mediated by cellular immunity, in which certain cytokines and Th1 cells have a critical role [9]. Understanding the mechanisms involved in this response, and in particular the function of the cytokine network involved in this disease, is of significant relevance to reach advances in the development of effective control and prevention [10].

## 2. Cytokines

Cytokines are molecules that mediate mainly the intercellular communication in the immune system, being produced by different cell types. Cytokines have pleiotropic and regulatory effects and participate in the host defense and in inflammatory and tissue reparation processes [11].

**Figure 1:**
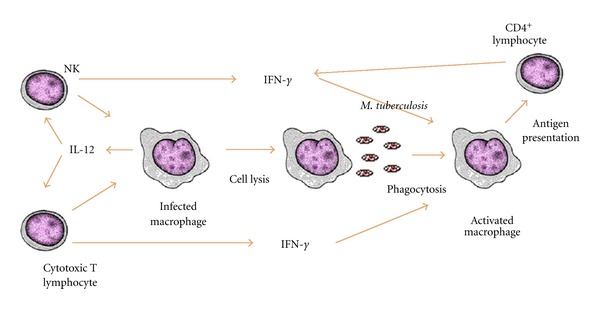
Initial protective response to *M. tuberculosis*-Th1 profile.

In tuberculosis, an effective and coordinated participation of different cytokines was already identified, such as interleukin-12 (IL-12), IL-23, IL-27, IL-18, IL-1, IL-7, and IL-15 [5]. An important aspect associated with the production of cytokines in MTB infections is the activation of macrophages in response to IFN-*γ* and TNF-*α* signaling [12]. Nonactivated macrophages are the usual habitat of MTB, which resists in the intracellular environment, blocking the phagosome fusion with the lysosome, thus avoiding its exposure to low pH and to reactive nitrogen intermediates (RNIs), important to its destruction [12]. IFN-*γ* activated macrophages transpose this blockage and form phagolysosomes expressing RNIs able to eliminate MTB in the infection sites [13]. The cord factor, the 19KDa lipoprotein, and other MTB components induce the production of IL-12 by macrophages, thereby mobilizing the Th1 cytokine pathway.  Figure 1 shows the initial immune protection to *M. tuberculosis.*

Several cytokines, including interleukin IL-12, IL-17, and IL-23, contribute to the host response to mycobacteria, improving the development of Th1 cells [14]. Among Th1 cytokines, IFN-*γ* and TNF-*α* were identified as the most important agents of the antimycobacterial cytokine cascade. This is due to the formation as well as the maintenance of the granuloma, which is mediated by TNF-*α* acting synergistically with IFN-*γ* in the activation of macrophages to produce effector molecules [15].

Recently, a new population of cells was identified and named Th17. These cells produce IL-17, IL-21, and IL-22 as signature cytokines [16, 17]. The IL-17 receptor is expressed in different organs including the liver, lung, and spleen, and different cell types are able to respond to IL-17, such as dendritic cells, macrophages, lymphocytes, epithelial cells, and fibroblasts. The responses induced by the IL-17 gene include expression of proinflammatory genes, chemokines, IL-6, IL-8, and antimicrobial proteins. Recent data suggest a superior and more complex role for these cells and their cytokines in different intracellular infections, including bacteria, fungi, and viruses in different mucosal surfaces [18]. Therefore, the balance between protection and Th17 cell-mediated pathology is the key in the definition of consequences in mucosal infections [19].

 Th17 cells also participate in the inflammatory response at an early mycobacterial infection; however, the production of IL-17 in the lungs is mainly immunosuppressive of IFN-*γ*. The protective potential role of Th17 cells during the early phase of infection with *M. tuberculosis* is unknown [20]. There are evidences on the role of IL-17 during mycobacterial infections. Pulmonary infection with BCG or *M. tuberculosis* stimulated the early secretion of IL-17 from the day 1 to 14 and sequentially the development of T cells secreting IFN-*γ*. Pulmonary infected IL-17 deficient mice with BCG showed a reduction in the delayed hypersensitivity responses, with a deficiency in granuloma formation in the lungs, suggesting that IL-17 is required for an efficient development of Th1 responses [20].

 The secretion of IL-23 is essential for the secretion of IL-17, and people with deficiency in the IL-12R*β*1 gene have low capacity to produce IL-23, and they have a lower production of IFN-*γ*. IL-12 is a cytokine that reduces the expression of IL-17, and this appears to show a self-regulation on inflammation. The balance between the secretions of IL-23/IL-17 and IL-12/IFN-*γ* appears to be essential for the regulation of inflammation in response to *M. tuberculosis* and other mycobacteria [21].

Among the cytokines that are being studied in response to *M. tuberculosis*, we can also emphasize interleukin-1. Interleukin-1 is necessary for the control of infection with *M. tuberculosis*, but the role of its two ligands, IL-1*α* and IL-1*β*, and its regulation *in vivo* are poorly understood. An important feature of IL-1 is its control on transcription, arranging the levels of transcription and signal transduction, as evidenced by the variety of immunopathologies and autoinflammatory diseases that occur in the absence of regulation of IL-1 [22, 23]. Little is known about the expression and processing of IL-1 in the context of infection with *M. tuberculosis in vivo*. The populations of cells that produce IL-1 during infection have not yet been characterized [24].

Guler et al. [25] investigated the role of IL-1*α* and IL-1*β* during chronic infection with *M. tuberculosis* and spontaneous reactivation of it in mice. Blockade of IL-1*α*, but not IL-1*β*, resulted in increased susceptibility to chronic infection with *M. tuberculosis*. When they neutralized IL-1*α* or IL-1*β* alone, they did not observe an increase in the reactivation of latent tuberculosis. The generation of antibodies neutralizing IL-1*α* and IL-1*β* simultaneously did not influence weight gain during reactivation, and they observed a slight increase in the lung bacilli count when compared to the immunized control group. Thus, their results suggested that IL-1*α* is the prime mediator of the IL-1RI-dependent and protective innate immune responses to *M. tuberculosis* in mice.

In a recent study the role of IL-1 in host resistance was demonstrated by inducing antibodies against this cytokine, which resulted in an increased mortality during chronic infection [25].

The granuloma is a typical structure of this disease, where we can find CD4 and CD8 T lymphocytes, B lymphocytes, macrophages, neutrophils, fibroblasts, and giant multinucleated cells. IFN-*γ*-producing CD4 T lymphocytes contribute to the generation of granulomas, besides being important costimulators to the adequate activation of CD8 T lymphocytes. The importance of CD4 T lymphocytes function is seen in patients with HIV, where the risk of TB increases with the decrease of the cells counting [26].

Many events mediated by cytokines are important to the establishment of immunity against MTB and the expression of host resistance [11].

**Response against M. tuberculosis**

CD4^+^ T cells exert regulatory activity on macrophage function, as well as cytolytic CD8^+^ lymphocytes. The effector function for the bacterial elimination is mediated by macrophages that are activated by cytokines derived from T lymphocytes, particularly IFN-*γ* and TNF-*α*.

## 3. Tumor Necrosis Factor (TNF-***α***)

The tumor necrosis factor (TNF, TNF-*α*) was originally characterized as a necrosis inductor in sarcomas *in vivo* [27]. TNF-*α* is a proinflammatory cytokine which exerts multiple biological effects. TNF-*α* expression is strictly controlled, since its superproduction can mediate damaging effects found in the septic shock such as arterial hypotension, disseminated vascular coagulation, and lethal hypoglycemia.

In the process of mycobacterial infection control, TNF-*α* seems to have a primordial role, acting upon a wide variety of cells. The main producing cells are activated macrophages, T lymphocytes, and dendritic cells [27–29]. This cytokine acts in synergy with IFN-*γ*, stimulating the production of reactive nitrogen intermediates (RNIs), thus mediating the tuberculostatic function of macrophages [30, 31]. TNF-*α* also stimulates the migration of immune cells to the infection site, contributing to the granuloma formation, capable of controlling the disease progression [32].

TNF-*α* blocking has dramatic effects on the progression of tuberculosis in experimental models. Neutralization of TNF-*α* in murine models results in tuberculosis aggravation or reactivation [32]. The excision of the TNF-*α* gene or its receptor results in deviant granulomas or fulminant acute tuberculosis [33, 34]. Studies have also revealed that TNF-*α* is expressed in MTB-infected tissues during the whole latent phase of infection [35], suggesting a contribution, with other cytokines like IFN-*γ*, in the control of the bacillus multiplication.

Increased levels of TNF-*α* are commonly detected in culture supernatants of peripheral blood mononucleated cells (PBMCs) from patients with pulmonary tuberculosis stimulated with mycobacterial antigens [10, 36, 37]. Moura [38] evaluating the immune response of patients prior to and after treatment noticed that patients with active pulmonary tuberculosis produced increased levels of TNF-*α*; however they did not observe significant difference in these cytokine levels after treatment, concluding that these results reinforce this cytokine's role at both the physiopathology and in the protective immunity of the disease.

A recent study investigated the role played by the nucleotide-binding oligomerization domain-containing protein 2 (NOD2) in the human alveolar macrophage innate responses and revealed that significant levels of IL-1*β*, IL-6, and TNF-*α* were produced after the recognition of the ligand with the muramyl dipeptide (MDP). Alveolar macrophage treatment with MDP has improved the control of intracellular growth of *M. tuberculosis*, activity associated with a significant production of TNF-*α* and IL-6 [39].

One of the most overwhelming lines of evidence of the protective effects of TNF-*α* is, perhaps, provided by the observation that patients with rheumatoid arthritis under treatment with TNF-*α* antagonists (monoclonal antibodies against TNF-*α* or TNF-*α* soluble receptors) have a significant increased risk of reactivating latent TB [40–42].

On the other hand, there is also evidence showing that TNF-*α* may be associated with immunopathological responses in tuberculosis, aforementioned also as the head mediator of the destruction of the pulmonary tissue [43]. Elevated levels of TNF-*α* are related to an excessive inflammation with necrosis and cachexy [44, 45].

Tumor necrosis factor (TNF-*α*) relative roles in MTb have been a subject of controversy. It was described that mycobacteria decreases the production of TNF in human PBMCs, skill which probably contributes to its ability to establish chronic infections [46]. Produced by macrophages, lymphocytes, neutrophils, and some endothelial cells, TNF-*α* coordinates the inflammatory response via induction of other cytokines (IL-1 and IL-6), and the recruitment of immune and inflammatory cells through the induction of chemokine and supraregulation of adhesion molecules. Experimental models have shown that TNF-*α* plays an important role not only in host response against *M. tuberculosis* but also in the immunopathology of tuberculosis [47].

TNF-*α* increases the capacity of macrophages to phagocytose and kill mycobacteria and stimulates apoptosis of macrophages, depriving bacilli of host cells and leading to death and presentation by dendritic cells of mycobacterial antigens [48]. *In vivo* TNF-*α* is required for the formation and maintenance of granulomas. Neutralization of TNF-*α* produced by mice chronically infected with *M. tuberculosis* specific monoclonal antibodies disrupts the integrity of granulomas, exacerbates infection, and increases mortality [49].

*M. tuberculosis* evolved and has developed mechanisms which interact and modulate the host immune response. Mycobacterium expresses surface antigens that can induce the production of IL-10 and IL-4, which typically have anti-inflammatory effects [50, 51]. The high expression of IL-4 has been implicated as a virulence factor, both for the anti-inflammatory ability and also for its apparent capacity to promote tissue damage in association with TNF-*α* [52]. These studies suggest that IL-4 (alone or jointly with TNF-*α*) may play a role in tissue destruction and/or cell death during infection by *M. tuberculosis*. TNF-*α* is one of the most powerful controlling factors for the recruitment of monocytes and is a potent inducer of cell death by apoptosis [53]. Necrosis, on the other hand, is associated with the lysis of the infected cell, the release of feasible *M. tuberculosis*, and damage to the surrounding tissues [54]. TNF-*α* is also a key cytokine involved in this event.

## 4. Interferon Gamma (IFN-*γ*)

Interferons (IFNs) are substances originally identified at cellular culture supernatants infected by virus and that appeared to interfere directly in the viral replication, hence its denomination [55]. Divided into two major types, type I IFNs are induced and act effectively in responses against viruses: IFN-*α* is secreted mainly by leucocytes, and IFN-*β* is produced by fibroblasts. Type II interferon, now referred to as IFN-*γ*, is synthesized mostly by T lymphocytes and NK cells after this cells activation with immune and inflammatory stimuli, rather than viral infection [56]. IFN-*γ* is the chief cytokine involved in the protective immune response against mycobacterial infection. It is produced primarily by CD4 and CD8 T lymphocytes and NK cells. It is also known that natural killer T cells (NKT) and *γδ* T lymphocytes, cells with a narrow repertoire of antigen recognition, can also produce IFN-*γ* in response to mycobacterial stimulation, displaying protection against *M. tuberculosis* infection both *in vitro* and *in vivo* [12].

The main function of IFN-*γ* is macrophage activation, rendering them able to exert its microbicidal functions. It operates also enhancing the antigen presentation through the induction of the expression of molecules from the major histocompatibility complex (MHC) class I and II and promoting the differentiation of CD4 T lymphocytes to the Th1 subpopulation [57–59]. IFN-*γ* induces the transcription of more than 200 genes in macrophages, including those for the production of antimicrobial molecules such as oxygen free radicals and nitric oxide, which represent one of the best effector mechanisms for elimination of *M. tuberculosis* [60]. However, some mycobacterial antigens, such as the 19 kDa lipoprotein, have the potential to mitigate the response of macrophages by blocking the transcription of subsets of genes responsive for IFN-*γ* [61, 62].

A series of clinical and experimental studies have demonstrated the importance of IFN-*γ* production in the control of tuberculosis [63–65]. Experiments in mice revealed that IFN-*γ* is an essential cytokine for macrophage activation and mycobacteria death in the intracellular environment. Cooper et al. [66] and Flynn et al. [67] have demonstrated that mice deprived from the IFN-*γ* genes have experienced fulminant infection by *M. tuberculosis*. 

Individuals with a deficiency in the IFN-*γ* receptor gene have shown to be extremely susceptible to mycobacterial infections [68]. The complete deficiency of IFN-*γ* receptor in humans is associated with increased severity in the course of infection, poor formation of granulomas, multibacillary lesions, and progressive infection [69]. Studies with individuals that presented genetic mutations in the IFN-*γ* receptor have also proven that they presented high susceptibility to atypical mycobacterial infections [70].

The interleukin-12 (IL-12)/interferon-*γ* (IFN-*γ*) axis is determinant to the generation of Th1 lymphocytes, activation of macrophages by T cells, and further elimination of bacteria. A series of mutations associated to these axis components were identified in humans: these include mutation in the IL-12R*β*1, IL-12p40, IFN-*γ*R2 genes, and the signal transducer and activator of transcription-1 (STAT-1). Most infections associated with these Mendelian disorders arise from the use of BCG or environmental mycobacteria. Nevertheless, some of the disorders are also associated with an increased susceptibility to *M. tuberculosis* (IFN-*γ*R2 and IL-12p40) [71, 72].

IL-12 enhances IFN-*γ* production by NK cells and expands antigen specific Th1 cells. Other cytokines such as IL-23, IL-18, and IL-27 are also important inducers of IFN-*γ*. About 20 days are enough to produce IFN-*γ* by Th1 lymphocytes, which results in its accumulation in the lungs and bacterial growth arrest [73]. IL-18, a cytokine produced by monocytes, macrophages, and dendritic cells, cooperates with IL-12 to induce IFN-*γ* production [61]. Studies clearly indicate that IL-18 contributes to protect against infection by mycobacteria [74, 75]. Moreover, IL-18 deficient mice when infected with *M. tuberculosis* present reduced levels of IFN-*γ* compared with normal mice, despite the standard levels of IL-12 [76]. 

Morosini and colleagues [77] emphasize through data that they found in their study the view that in humans, at least at certain stages of pulmonary tuberculosis, there is a differential compartmentalization of IFN-*γ* and of the regulatory cytokine IL-12 and IL-10, where the protection factor associated with the secretion of IL-12 is present in the lungs and the component associated with immunosuppressive IL-10 secretion is predominant in peripheral blood. Furthermore, their results indicate a more critical role for IL-18 in the host response to *M. tuberculosis* in humans, suggesting that IL-18 may act as a factor for induction of IFN-*γ* in the lungs, whereas one can have immunoregulatory activity on peripheral circulation [77].

Studies report that patients with less severe forms of pulmonary tuberculosis have a predominance of Th1 cytokines such as IFN-*γ*, whereas the increase in IL-4 levels, a Th2 type cytokine, is related to the disease severity [78, 79]. Torres et al. [80] studied the immune response of patients' PBMCs with active tuberculosis and their healthy household contacts in response to the 30-KDa antigen from *M. tuberculosis*. Their results demonstrated a defect in the IFN-*γ* production by patients in response to the investigated antigen and a strong response to this antigen by the healthy communicants' cells, suggesting a protective role of IFN-*γ* in those individuals.

After inhalation and subsequent infection with *M. tuberculosis *in the lungs, dendritic cells infected with the bacilli migrate to the regional lymph node, which occurs, on average, around 14 days after infection, initiating the activation of T cells [81]. A model study used dendritic cells infected with *M. tuberculosis* inoculated intratracheally in the lung and as a result found that dendritic cells exposed to *M. tuberculosis* prior to inoculation are better in migrating to the lymph node and in T cell activation [82, 83]. These findings, in association with information about the secretion of cytokines and activation of the populations of CD4^+^ T cells, indicate that different subtypes of CD4^+^ T cells involved in protection in tuberculosis are activated in the initial phases of infection and produce cytokines classically considered immune protectors such as IL-2, IFN-*γ* and TNF-*α* [84, 85].

There is evidence that CD4^+^ T cells may contribute both to the control of *M. tuberculosis* as well as of immunopathology, contributing to morbidity and mortality in tuberculosis disease. Reference [86] quantified the variation of IFN-*γ*/IL-17 in response to specific antigens of *M. tuberculosis* in patients with a positive PPD test and healthy individuals and observed a large variation in the amounts of IL-17 and IFN-*γ* secreted in response to the various antigens used.

## 5. Interleukin-10 (IL-10)

Due to its ability to inhibit the T lymphocyte production of cytokines, IL-10 was originally described as a cytokine synthesis inhibitory factor (CSIF) [86]. Subsequent studies have demonstrated that IL-10 could also inhibit Th1 and Th2 subpopulations *in vitro* [87, 88]. IL-10 acts inhibiting the production of pro-inflammatory cytokines (IFN-*γ*, TNF-*α* and IL-12) and the action of antigen presenting cells, blocking the activation of T lymphocytes through the inhibition of expression of MHC class II molecules [89, 90]. Therefore, it has an immunoregulatory function [91].

IL-10 is produced by macrophages and T lymphocytes during *M. tuberculosis* infection. Unlike TNF-*α* and IFN-*γ*, IL-10 is considered primarily an inhibitory cytokine, important to the adequate balance between inflammatory and immunopathological responses. However, the increase in IL-10 levels appears to support the mycobacterial survival in the host. Mice with defective IL-10 exhibit an increase in the antimycobacterial immunity [92]. IL-10 reduces the protective response to MTB in the CBA mice strain, in which IL-10 is produced by phagocytes in the interior of the pulmonary lesion and where a reduction in the TNF and IL-12p40 expression can be observed [93]. IL-10 is also able to induce the reactivation of tuberculosis in animals [94].

IL-10 is increased in samples obtained from patients with TB, and a higher capacity of IL-10 production is associated with an increase in the disease incidence. In human tuberculosis, IL-10 production is higher in anergic patients, suggesting that *M. tuberculosis* induces IL-10 production, suppressing an effective immune response [95]. Macrophages from patients suffering from tuberculosis are suppressed *in vitro*, and the inhibition of IL-10 reverts partially this suppression [90]. In another study, IL-10 was capable of directly inhibiting the responses of CD4 T lymphocyte from donors with latent TB and also reduced the expression of MHC class I and II, CD40, B7-1, and B7-2 of monocytes infected with MTB [96].

Lowering the protective cellular immune response is the *M. tuberculosis* aim to survive in the host. IL-10 and other inhibitory mediators of the inflammatory response (TGF-*β*RII, IL-1Rn e IDO) are detected in the sputum samples of patients with TB, whereas 30 days after treatment their level decreases considerably, while an increase in the Th1 response is observed [97].

In some human populations, an increase in IL-10 expression was identified, being possible to correlate it with an inefficiency in the BCG (Bacillus Calmette-Guérin) vaccination [98]. The analysis of the IL-10 gene polymorphisms involved in the development of infectious diseases suggests that this polymorphism has a critical role in the immunity and progression of inflammation. The increase in IL-10 production can, in particular, suppress the immune response and promote progression of the disease [99]. 

Regarding the immunopathogeny of TB, it is unquestionable the immunosuppressor role presented by IL-10 [100, 101]. Nonetheless, some studies have not detected increased levels of this cytokine in PBMCs from patients with active TB in response to mycobacterial antigens [102]. 

## 6. Cytokines in Household Tuberculosis Contact Cases

The study of household contacts of TB cases is of essential importance to the programs for combat and control of tuberculosis, in other words, the epidemiologic surveillance of household contacts as a means for early diagnosis of TB cases and the decrease of the spread of the disease [103].

Some studies assessed the cytokine profile in groups of healthy contacts individuals. From these works, it is important to mention Demissie [104], who conducted a study comparing the immune response of infected individuals in the latent stage with TB patients. The results demonstrated that TB patients presented a low production of IFN-*γ* and IL-2 cytokines when compared to individuals with latent infections. This suggests that the control of TB in the latent stage is not only associated with increased expression of Th1 cytokines, but also with the suppression of IL-4 activity [104]. Later, the same group [105] compared the expression of IL-4, IL-4*δ*2, and IFN-*γ* in the peripheral blood of household contacts of TB patients presenting positive sputum. The results demonstrated that the expression of IL-4 was slightly higher in household contacts when compared to the controls from the community. However, when the household contacts were divided into groups with or without immunological signs of infection with *M. tuberculosis,* the expression of IL-4 was clearly elevated in the positive ESAT-6 (signal transducer and activator of transcription 6, an MTB antigen) group and the expression of Th1 cytokines such as IFN-*γ* was low. Thus, they suggest that a strong response to the antigen ESAT-6 in individuals exposed to *M. tuberculosis* correlates with a low expression of IFN-*γ* and higher expression of IL-4 and that possibly this profile is associated with a poor prognosis [105]. The results founded by our group [106] demonstrated that individuals with or without a previous history of tuberculosis and exposed to *M. tuberculosis* showed a Th1 (TNF-*α* and IFN-*γ*) and Th2 (IL-10 and TGF-*β*) profile of cytokines, similar to that found by Demissie [105], with an IFN-*γ* production relatively low when compared to IL-10. These cytokines would be involved in shifting the state of latency to the stage of clinical tuberculosis [106].

The presence of high levels of IL-10 in the plasma of household contacts was unexpected for the group of [107], since they have also found high levels of IL-10 in the studied patients. However, there are few reports in the literature on the production of IL-10 by household contacts of patients with tuberculosis [107]. According to [107], these levels are due to stimulation of mycobacterial antigens that induce this cytokine production by mononuclear cells. Moreover, we can suggest that IL-10 was involved in the natural defense that goes against excessive proinflammatory responses generated by TNF-*α*. Therefore, the simultaneous presence of IL-10 and TNF-*α* in household communicating TB patients might be beneficial to these individuals [105]. IL-10 may be required to modulate proinflammatory effects in patients and in healthy household tuberculosis individuals.

Table 1 summarizes the main cytokines, associated studies, and references described in this paper.

## 7. Final Thoughts

The host resistance against infection with *M. tuberculosis* starts with the innate immunity, involving the interaction of the bacillus with macrophages and dendritic cells. Little is known about the transition between the initial control of infection and the establishment of latent infection, which is largely due, in part, to the lack of appropriate animal models [108].

Although there are still no conclusive studies about a clear dichotomy between Th1 versus Th2 response, involving protective immunity and disease susceptibility, respectively, we can conclude that the knowledge of Th1 and Th2 responses helps to elucidate the immune protection profile of the host against *M. tuberculosis*.

## Figures and Tables

**Table 1:** Studies of cytokines associated to *M. tuberculosis* infection.

Cytokine	Tuberculosis study	References
TNF-*α*	Studies in murine models	[32–35, 44]
	Studies in patients with pulmonary tuberculosis	[36–39]

IFN-*γ*	Studies in murine models	[45, 66, 67, 76]
	Clinical studies	[63–65, 77–80, 85]
	Genetic studies	[68–72]

IL-10	Studies in murine models	[92–94]
	Studies in patients with tuberculosis	[95–102]

Other cytokines	Studies in tuberculosis	[14, 16–21, 24, 25]
